# Small‐Molecule and Peptide Inhibitors of the Pro‐Survival Protein Mcl‐1

**DOI:** 10.1002/cmdc.201500497

**Published:** 2015-12-23

**Authors:** Andrew M. Beekman, Lesley A. Howell

**Affiliations:** ^1^ School of PharmacyUniversity of East AngliaNorwich Research ParkNorwichNorfolkNR4 7TJUK

**Keywords:** Bcl-2, Mcl-1, protein–protein interactions, small-molecule inhibitors, stapled peptides

## Abstract

The ability of protein–protein interactions to regulate cellular processes in both beneficial and detrimental ways has made them obvious drug targets. The Bcl‐2 family of proteins undergo a series of protein–protein interactions which regulate the intrinsic cell‐death pathway. The pro‐survival members of the Bcl‐2 family, including Bcl‐2, Bcl‐x_L_, and Mcl‐1, are commonly overexpressed in a number of human cancers. Effective modulators of members of the Bcl‐2 family have been developed and are undergoing clinical trials, but the efficient modulation of Mcl‐1 is still not represented in the clinic. In addition, Mcl‐1 is a major cause of resistance to radio‐ and chemotherapies, including inhibitors that target other Bcl‐2 family members. Subsequently, the inhibition of Mcl‐1 has become of significant interest to the scientific community. This review covers the progress made to date in modulating the activity of Mcl‐1, by both stapled peptides and small molecules. The development of peptides as drug candidates, and the advancement of experimental and computational techniques used to discover small molecules are also highlighted.

## 1 Introduction

Protein–protein interactions (PPIs) control many important physiological processes within human cells.^1^ The ability of specific proteins to interact with high specificity and affinity has been observed in processes both beneficial and detrimental to health. Additionally, changes in these interactions can lead to specific cellular processes malfunctioning, potentially resulting in biologically undesirable effects, for example the ability of cancer cells to avoid apoptosis. The modulation of PPIs has the potential to return physiological processes to healthy states, and as such there is much interest in the development of agents which will allow such control.^2^ This task, however, is not a trivial one, with PPIs historically being considered “undruggable” because of their large and shallow interfaces.^3^ In recent years new approaches have been developed to effectively target PPIs using both computational and experimental approaches.3b, 4 This has resulted in the preparation of a number of small‐molecule modulators, with more than 12 candidates currently in clinical trials.^5^

Proteins of the Bcl‐2 family regulate the intrinsic mitochondrial cell death pathway with the family consisting of both pro‐ and anti‐apoptotic members.^6^ Over the past decade there has been significant interest in the PPIs of the Bcl‐2 family due to the role they play in apoptosis (we refer the reader to reference 7b for a detailed discussion of the Bcl‐2 family proteins).^7^ The ability of cancer cells to avoid apoptosis plays a large role in tumour progression and drug resistance.7a The Bcl‐2 family consists of both pro‐apoptotic proteins, anti‐apoptotic proteins and regulators. The pro‐apoptosis proteins include BAX and BAK, and are triggered through their interaction with activating regulators BIM, BID, and PUMA.^8^ The interaction between the pro‐apoptotic proteins and regulators is a key element of cell death. Anti‐apoptotic proteins such as Bcl‐2, Bcl‐x_L_ and Mcl‐1 prevent activation of BAX or BAK by binding the activating regulators or the pro‐apoptosis proteins themselves, keeping cells alive.^9^ This prevents BAX and BAK from oligomerising and puncturing the outer mitochondrial membrane resulting in the release of cytochrome C into the cytoplasm. This is a rapid and irreversible process which activates downstream caspases and is the point of no return for the cell. The anti‐apoptotic proteins are modulated by sensitiser regulators including BAD and NOXA, which do not activate BAX or BAK, but preferentially bind to Bcl‐2, Bcl‐x_L_ and Mcl‐1, deactivating them. The overexpression of anti‐apoptotic proteins is commonly observed in a number of human cancers, resulting in cell survival.^10^ These proteins share regions of homology named Bcl‐2‐homology (BH) domains. The regulating proteins are BH3‐only proteins (meaning they only possess homology in the BH3 region) and are induced through cellular stress death signals. These proteins bind through hydrophobic and electrostatic interactions between the BH3 region and the binding groove formed by the BH1, BH2 and BH3 regions of the pro‐ and anti‐apoptotic proteins.^11^ Candidates are already in clinical trials which inhibit Bcl‐2 and Bcl‐x_L_ and have been shown to induce apoptosis as BH3 mimetics (see reference 12 for a recent review). However, the inability to inhibit all anti‐apoptotic proteins, in particular Mcl‐1, has been shown to result in drug resistance.7a Despite being one of the most frequently amplified genes in cancer and being a major factor in resistance to chemotherapy, Mcl‐1 has proven the most elusive, with no candidates currently in clinical trial. The pro‐survival Bcl‐2 family proteins sequester the α‐helical BH3 domain of the pro‐death Bcl‐2 family, in the binding groove created by the BH1‐3 domains. However, Mcl‐1 differs from the other pro‐survival members, possessing a more electropositive binding groove,^8^ with a number of different residues.^13^ Additionally, the Mcl‐1 groove has been shown to be more rigid than the other pro‐apoptosis members,^14^ making specific modulators difficult to obtain.

Several examples of small molecules which modulate Mcl‐1 have been reported, with a variety of reviews addressing patented compounds,^15^ compounds in clinical trials^8^ and pan‐Bcl‐2 inhibitors.^16^ Most recently, Belmar and Fesik presented an excellent review of Mcl‐1 binders which are known to be BH3 mimetics.^17^ The scope of this review will target a comprehensive overview of known Mcl‐1 binders which have been demonstrated to inhibit the PPIs of Mcl‐1 with the pro‐apoptotic and regulating proteins from the Bcl‐2 family, complementing the work which has already been presented before us.

## 2 Stapled Peptides

In general terms there are two major classes of approved drugs: small molecules and protein therapeutics.^18^ A small‐molecule inhibitor of a PPI is generally much smaller than the protein it is inhibiting, therefore limiting the number of interactions it can make. However, the presence/requirement of a hydrophobic groove or pocket enables the design of small molecules capable of inhibiting the protein of interest,^19^ and small‐molecule inhibitors of Mcl‐1 are discussed in detail below. By contrast, protein therapeutics are much larger and have a greater surface area in which to make contact with the target. Therefore, they do not need such defined binding pockets and can effectively bind to flatter, shallower surfaces which are not necessarily hydrophobic. However, this class of drug is unable to cross the cell membrane, so is therefore limited to extracellular targets. An alternative and attractive option for stabilising or disrupting PPIs are peptides.^20^ However, in vivo their efficacy is compromised due to a loss in secondary structure as well as poor cellular uptake and susceptibility to proteolysis. A promising synthetic approach to overcome these limitations is to “staple” the peptide to fix its orientation.^21^ In addition to being a potential new class of therapeutics capable of inhibiting the Bcl‐2 proteins, they may also prove useful chemical biology tools for probing these key proteins and furthering our understanding of the processes governing apoptosis.

**Figure 1:**
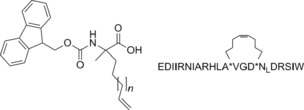
Fmoc‐protected amino acid building block and the BID SAHB_A_ hydrocarbon‐stapled peptide.

Peptide stapling was first introduced by Verdine and co‐workers in 2000^22^ who then went on to employ the concept to identify peptide‐based inhibitors of Bcl‐2 proteins designed on the structure of the BH3 domain of the BID protein (Table 1, Figure 1), so called “stabilised α‐helix of BCL‐2 domains” (SAHBs).^20^ BID SAHB_A_ was able to specifically activate the apoptosis pathway in leukaemia cells and inhibit the growth of leukaemia xenografts in vivo. Further characterisation of the peptide demonstrated it bound to and activated BAX directly.^23^ Following this pioneering work a variety of BH3‐only peptide domains were used to synthesise stapled peptides capable of modulating apoptosis.^24^

In 2010 Loren Walensky′s group identified the first stapled peptide capable of selectively inhibiting Mcl‐1 (Table 1).^25^ The development of such selective inhibitors is a major challenge due to the very subtle differences between the Bcl‐2 family proteins in terms of their hydrophobic grooves. A series of stapled peptides based on the BH3 domains of both pro‐ and anti‐apoptotic Bcl‐2 proteins were designed and assessed for their ability to inhibit Mcl‐1 by fluorescence polarisation (FP) assay. Those that bound Mcl‐1 with a high affinity were further evaluated against a panel of anti‐apoptotic targets and the Mcl‐1 BH3 helix was found to be a potent and selective inhibitor of Mcl‐1 capable of sensitising cells to caspase‐dependent apoptosis. Alanine scanning, site‐directed mutagenesis and staple scanning were used to determine the key binding sites and specificity determinants as well as the optimal helicity. Interestingly, X‐ray crystallography and mutagenesis studies revealed the hydrocarbon staple itself is capable of making additional hydrophobic interactions, which along with the enhanced α‐helicity may be responsible for the enhanced binding affinity of Mcl‐1 SAHB_D_. This was later supported by molecular dynamics simulations.^26^ The Walensky group then went on to use these stapled peptides, specifically Mcl‐1 SAHB_A_ as a screening tool to identify small molecules (described below).^27^

**Table 1:** SAHB peptides reported by Verdine^[20]^ and Walensky.^[25]^

Compound	Sequence^[a]^	Helicity [%]^[b]^	*K*_d_ [nm]
BID BH3	EDIIRNIARHLAQVGDSN_L_DRSIW	15.7±0.3	269^[c]^
BID SAHB_A_	EDIIRNIARHLA*VGD*N_L_DRSIW	87.5±0.3	38.8^[c]^
BID SAHB_A(G→E)_	EDIIRNIARHLA*VED*N_L_DRSIW	77.8±0.6	483^[c]^
BID SAHB_B_	EDIIRNI*RHL*QVGDSN_L_DRSIW	85.5±1.3	n.d.
BID SAHB_C_	EDIIRNIA*HLA*VGDSN_L_DRSIW	59.7±6.5	n.d.
BID SAHB_D_	EDIIRNIAR*LAQVGD*N_L_DRSIW	35.6±1.8	n.d.
Mcl‐1 BH3	KALETLRRVGDGVQRNHETAF	18	245±29^[d]^
Mcl‐1 SAHB_A_	KALETLR*VGD*VQRNHETAF	62	43±16^[d]^
Mcl‐1 SAHB_B_	KAL*TLR*VGDGVQRNHETAF	100^[e]^	18±4^[d]^
Mcl‐1 SAHB_C_	KALETLRRV*DGV*RNHETAF	81	>1000^[d]^
Mcl‐1 SAHB_D_	KALETLRRVGDGV*RNH*TAF	91	10±3^[d]^
Mcl‐1 SAHB_E_	KALETLRRVGDGVQR*HET*F	68	33±10^[d]^

[a] ✶: Indicates location of hydrocarbon staple. [b] Determined by circular dichroism. [c] Determined by a Bcl‐2 FP assay; 95 % CI BID BH3 33.5–44.9 nm, BID SAHB_A_ 244–297 nm, BID SAHB_A(G→E)_ 434–536 nm. [d] Determined by an Mcl‐1 FP assay; data are the mean±SD performed in at least triplicate; n.d.: not determined. [e] Exceeds calculated ideal value for undecapeptide standard.

In 2012 Lin and co‐workers reported the design of proteolytically stable and cell permeable peptide‐based inhibitors of Mcl‐1 (Table 2).^28^ Rather than employing a hydrocarbon cross‐link as used by Verdine and Walensky, a bis‐aryl staple was incorporated into a Noxa peptide which binds to Mcl‐1 with high affinity and selectivity. Two solvent‐exposed *i* and *i*+7 residues were replaced with d‐ or l‐cysteine and the peptide subjected to 4,4′‐bis(bromomethyl)biphenyl (Bph)‐mediated cross‐linking.^29^ The resulting cross‐linked peptides possessed enhanced inhibitory activity when compared to the parent Noxa peptide whilst still maintaining selectivity. However, the compounds were inactive in cellular assays due to poor uptake. Replacement of three solvent exposed, positively charged residues (Arg6, Arg14, and Lys16) improved affinity (roughly twofold increase) but more importantly led to increased cellular activity. N‐Methylation of both N‐terminal alanine residues led to improved activity in both the FP assay as well as in the cell viability assay. Fluorescence‐activated cell sorting (FACS) analysis of fluorescently tagged versions of the peptides confirmed increased cellular uptake and confocal microscopy showed the peptides were predominantly localised in the cytosol. Finally the stapled peptides were shown to possess enhanced stability toward proteases.

**Table 2:** Stapled Noxa peptides described by Lin and co‐workers.^[28]^

Compound	Sequence^[a]^	*K*_i_ [nm]^[b]^	Cell Viability [%]^[b]^
Noxa	AAQLRRIGDKVNLRQKLLN	648±128	97.6±0.9
Noxa 1	AAC′LRRIGDC′VNLRQKLLN	10±1	98.6±4.0
Noxa 2	AAc′LRRIGDC′VNLRQKLLN	54±14	100.3±0.2
Noxa 3	AAc′LRAIGDC′VNLRQKLLN	23±8	85.9±2.2
Noxa 4	AAc′LRAIGDC′VNLAQKLLN	28±11	72.9±3.2
Noxa 5	AAc′LRAIGDC′VNLAQALLN	29±4	44.3±0.2
Noxa 6	A_m_Ac′LRRIGDC′VNLRQKLLN	32±3	87.3±2.8
Noxa 7	A_m_A_m_c′LRRIGDC′VNLRQKLLN	22±4	80.5±4.7
Noxa 8	A_m_A_m_c′LRAIGDC′VNLAQALLN	22±8	34.8±0.5

[a] C′=Bph‐linked l‐Cys, c′=Bph‐linked d‐Cys, A_m_=*N*‐methylalanine. [b] Data are the mean±SD, *n*=3.

In 2014, Lin went on to report the effects of the flexibility/rigidity as well as the length of the cross‐linker;^30^ suggesting a more flexible cross‐linker may allow the peptide to adopt a more favourable/active conformation when bound to its protein partner. A series of aryl and vinylaryl cross‐linkers with varying linker length, rigidity and hydrophobicity were synthesised (again using the Noxa BH3 peptide) and the compounds evaluated for inhibitory Mcl‐1 activity using a competitive FP assay. Analogues containing a 6,6′‐bis(bromomethyl)‐3,3′‐bipyridine (Bpy) or *p*‐phenylene‐3,3′‐bis(allylbromide) cross‐linker appeared to have the highest helicity which translated into the highest inhibitory activity in the FP assay, whereas cellular uptake correlated with hydrophobicity with the analogues containing the Bph staple or a 3,3′‐bis(bromomethyl)biphenyl variant showing the most efficient cellular uptake.

Taken together these results suggest the stapling of peptides is a promising new approach to targeting PPIs, specifically Mcl‐1 and other Bcl‐2 family proteins. The bioactive conformation of the peptide is maintained whilst overcoming the drawbacks of using peptides as drug molecules; careful positioning and choice of staple can result in a high‐affinity binder with improved cellular uptake and stability.

## 3 Small‐Molecule Inhibitors

### 3.1 Antimycin A


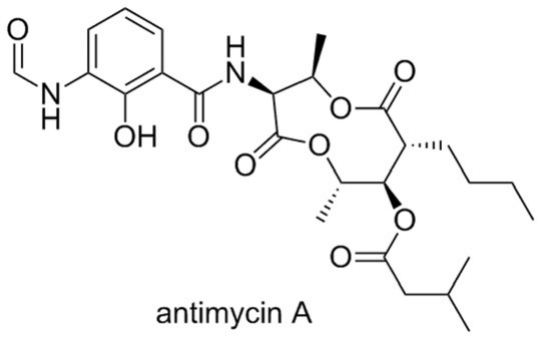
In 2001 Tzung and co‐workers reported that antimycin A, a natural product antibiotic isolated from a *Streptomyces* sp., markedly increased the apoptosis induced cell death in cell lines possessing Bcl‐x_L_ associated multidrug resistance.^31^ Through docking studies, they showed that the binding groove of the Bcl‐2 family proteins was occupied by antimycin, confirmed by their fluorescence assays on Bcl‐2 and Bcl‐x_L_. Reed and co‐workers subsequently demonstrated the ability of antimycin A to competitively bind to Mcl‐1 at similar concentrations to Bcl‐2 and Bcl‐x_L_ (IC_50_=2.51 μm, FITC‐Bid BH3‐only peptide).^32^

### 3.2 BH3I‐1


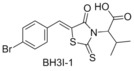
Yuan and co‐workers highlighted three compounds from a competitive FP binding assay of the Bcl‐x_L_‐BH3 site and Bak. These three compounds, titled BH3I‐1 (BH3 Inhibitor‐1), BH3I‐1’ and BH3I‐2, induced apoptosis in JK cells, showing the characteristic features of over‐expression of pro‐apoptotic Bcl‐2 family proteins.^33^ Yuan showed that BH3I‐1 acts by preventing the heterodimerisation of the pro‐apoptotic and anti‐apoptotic Bcl‐2 proteins, and identified the binding site through NMR studies. Reed demonstrated that BH3I‐1 is a competitive Mcl‐1 binder with an IC_50_ of 2.17 μm (FITC‐Bid BH3‐only peptide).^32^

### 3.3 BH3M6


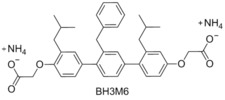
In 2002 Hamilton and co‐workers synthesised a number of compounds which were designed to mimic the binding residues of the Bcl‐2 family proteins.^34^ Using the crystal structure of Bak/Bcl‐2 they identified a number of hydrophobic residues which were shown to participate in binding by alanine scanning. This structure guided design resulted in the preparation of BH3M6. Hamilton demonstrated that BH3M6 was able to inhibit the binding of Bak and Bcl‐x_L_ with a *K*_d_=1.89 μm. In 2011 Sebti and co‐workers demonstrated that BH3M6 inhibits the binding of Mcl‐1 to Bax and Bim in a dose‐dependent manner in HEK293T and A549 cells.^35^ Sebti also demonstrated that BH3M6 can induce apoptosis by acting as a pan‐inhibitor and disrupting the formation of Bcl‐x_L_/Bim, Bcl‐2/Bim and Mcl‐1/Bim heterodimers.

### 3.4 YC137


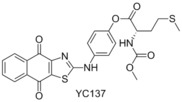
In 2001 Wang and Yang performed a virtual screen of 206 876 compounds based on an NMR structure of Bcl‐2, and then performed a subsequent in vitro assay which highlighted 35 of these structures.^36^ In 2004 Wang proceeded to report the exploration and elaboration of these structures, which highlighted YC137 as a potent Bcl‐2 inhibitor.^37^ Reed again showed this compound to be a strong Mcl‐1 binder, demonstrating an IC_50_ of 2.47 μm in their FP assay (FITC‐Bid BH3‐only peptide).^32^ Despite the competitive binding ability, and the recognised selectivity of YC137 for Bcl‐2 family members, the structure has received little attention in the literature regarding the activity toward Mcl‐1.

### 3.5 EGCG


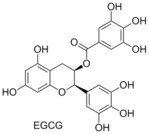
There has been much attention in the literature regarding the anticancer ability of compounds found commonly in green and black tea.^38^ In 2003 Pellecchia and co‐workers reported the examination of a number of natural compounds found in significant quantities in green and black tea, with a particular interest in their ability to bind the Bcl‐2 family. They showed that (−)‐epigallocatechin gallate (EGCG) had binding affinities in the nanomolar range for Bcl‐2 (*K*_i_=490 nm) and Bcl‐x_L_ (*K*_i_=335 nm, FITC‐BAD‐BH3‐only peptide competitive binding assay).^39^ Computational docking studies revealed that EGCG bound to the BH3 domain. Reed and co‐workers demonstrated that EGCG was a pan‐inhibitor of the Bcl‐2 family.^32^ Recently, it has been shown that in several cancer cell lines (786‐O renal cell carcinoma, HNSCC, Pc‐3 and LNCaP), EGCG binds upstream of the Bcl‐2 family, resulting in downregulation of the anti‐apoptosis proteins.^40^

### 3.6 (−)‐Gossypol (AT‐101)


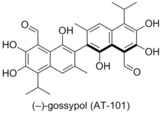
In 2002 Wang and co‐workers patented the use of (−)‐gossypol (AT‐101) as a Bcl‐2 family antagonist, after performing a structure‐based database screen.^41^ Gossypol was shown to bind to Bcl‐x_L_ and Bcl‐2 with high affinity (320 nm and 480 nm respectively),^42^ as well as to Mcl‐1 (180 nm, FITC‐Bad‐BH3‐only peptide). Subsequently, gossypol was advanced to clinical trials as a small‐molecule inhibitor of Bcl‐x_L_, Bcl‐2 and Mcl‐1.^43^ In 2004 Bradford and co‐workers reported that (−)‐gossypol was acting as a BH3 domain mimetic, making it a pan inhibitor of the Bcl‐2 family of proteins.^44^ Subsequently, (−)‐gossypol was shown to delay the onset of androgen‐independent prostate cancer in vivo,^45^ chemosensitise prostate cancer cells (PC‐3) to docetaxel both in vitro and in vivo, and was proven to increase the availability of the pro‐apoptotic proteins Puma and Noxa.^46^

### 3.7 TW‐37


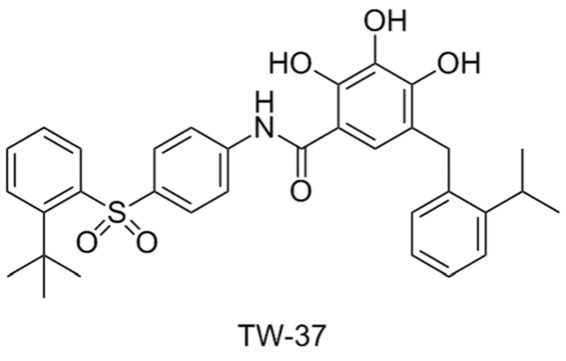
In 2006 Wang et al. reported the structure‐based design of a new series of Bcl‐2 family binders, based on the exploration of the structure activity relationship of gossypol.^47^ The Bcl‐2 protein was targeted, with computational docking calculations, to examine which functional groups played the largest role in gossypol′s affinity to Bcl‐2. It was shown that the polyphenol system was of high importance, and the hydrophobic section of the molecule could be extended to better fit into the binding pocket. After a series of iterations Wang and co‐workers prepared TW‐37, which showed a high affinity for Bcl‐2 (*K*_i_=290 nm) and Mcl‐1 (*K*_i_=260 nm), but lower affinity for Bcl‐x_L_ (*K*_i_=1110 nm, FAM‐Bid‐BH3‐only peptide competitive binding assay). Wang and co‐workers went on to show that TW‐37 inhibits cell growth in the prostate cancer cell line PC‐3 with an IC_50_ value of 200 nm, and induces apoptosis in 89.5 % of cells at 5 μm.^47^

In 2007 Mohammad and co‐workers demonstrated that TW‐37 resulted in the inhibition of cell proliferation of lymphoma cells (WSU‐DLCL_2_ 290 nm) and induced apoptosis in 71.4 % of cells at 400 nm.^48^ Furthermore, they showed that TW‐37 enhances the efficacy of the four drug combination cyclophosphamide‐doxorubicin‐vincristine‐prednisolone (CHOP) in mice models, displaying significant decrease in tumour weight relative to CHOP alone, TW‐37 alone or the control.^48^ These results were similarly reflected in the examination of B‐cell tumour cell lines by Katib and co‐workers.^49^

### 3.8 Apogossypolone (ApoG2)


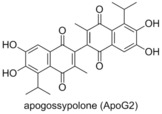
Apogossypolone (ApoG2) is a gossypol derivative prepared by Mohammad and co‐workers in 2008.^50^ It was shown to possess low nanomolar affinity for Bcl‐2 (*K*_i_=35 nm) and Mcl‐1 (*K*_i_=25 nm, competitor not published). Examination of the activity of apogossypolone in Follicular Lymphoma cells (WSU‐FSCCL) showed an IC_50_ of 109 nm, and decreased cell numbers in a fresh lymphoma sample. Apogossypolone was demonstrated to prompt apoptosis, with an activation of the apoptosis inducing factor, suggesting that apogossypolone was acting as a BH3 mimetic.

### 3.9 Obatoclax


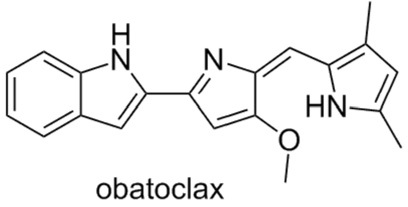
Obatoclax was developed by Gemin X,^15^ a developmental program focussed on modulating the anti‐apoptototic proteins of the Bcl‐2 family.^51^ It was demonstrated to be a pan‐Bcl‐2 inhibitor by Reed and co‐workers,^32^ but was shown by Shore to be more potent than the original FP assay suggested, due to the low solubility in aqueous medium.^51^ Shore went on to display that obatoclax overcomes resistance to apoptosis conferred by Mcl‐1, restoring sensitivity to the known Bcl‐2 antagonist ABT‐737, by increasing the presence of Bim in the cell. Additionally, obatoclax displayed single‐agent antitumour activity in multiple standard mouse tumour models.^51^ Obatoclax was shown to bind tightly to Mcl‐1 by inducing a histidine (His252) side chain hydrogen bond, in a narrow groove of the binding site, which is conserved in several Bcl‐2 family members.^52^ Obatoclax was also found to increase the activity of cisplatin in both resistant and sensitive cell lines, overcoming platinum resistance in the former and restoring mitochondrial apoptosis.^53^ Obatoclax has been investigated in several phase I/II clinical trials, both as a single agent and in combination. However to date, the data available suggests a low therapeutic activity of obatoclax.^12^

### 3.10 S1


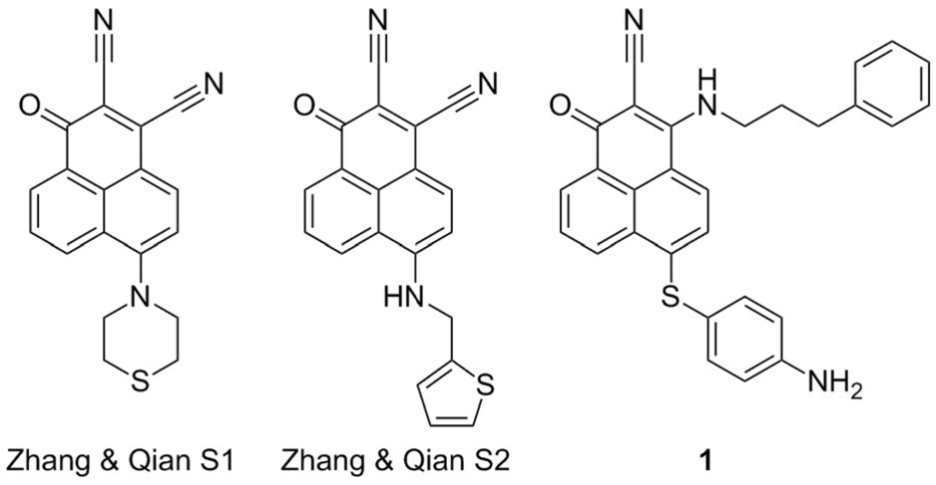
Zhang and Qian reported in 2007 the design of compounds termed S1 and S2, and demonstrated them to be cytotoxic compounds.^54^ Flow cytometry showed that S1 was inducing apoptosis in vitro, and subsequent analysis showed that this was also the case in vivo. Examining the process upstream, Zhang showed that S1 bound to Bcl‐2, via NMR‐based binding assays, and demonstrated through computational docking that S1 and S2 were binding in the BH3 domain. S2 was shown to have an IC_50_ of 1.3 μm to HL‐60 cells and S1 to have an IC_50_ of 2.8 μm, reproducing their computational predictions.54a Zhang reported in 2011 that S1 was also an Mcl‐1 inhibitor, preventing the Mcl‐1/Bak dimerisation, claiming it to be the first reported “authentic BH3 mimetic”.^55^ In 2013 Zhang demonstrated that S1 shows activity toward a variety of leukaemia cell lines, including acute lymphoblastic leukaemia, acute myeloid leukaemia, chronic lymphoblastic leukaemia and chronic myeloid leukaemia.^56^ While S1 inhibited Mcl‐1 and Bcl‐2 as expected, the protein Bcl‐2 did provide resistance to S1, and the ratio of these proteins allowed for the prediction of the cytotoxicity of S1. Elaboration of S1 and S2 led to product **1**, which showed a 9‐ to 35‐fold higher affinity for Mcl‐1, Bcl‐2 and Bcl‐x_L_ than S1, displaying IC_50_ values of 10, 20, and 18 nm, respectively (ELISA).^57^

### 3.11 Meiogynin A derivatives


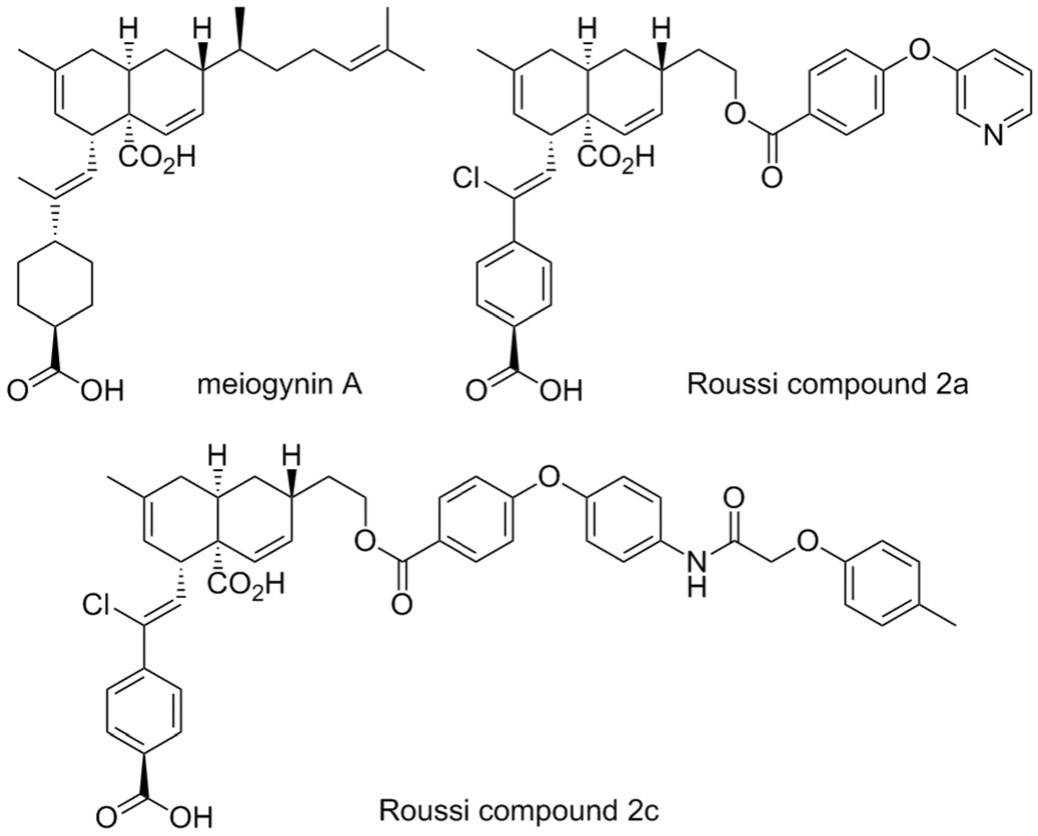
In 2009 Gueritte and co‐workers isolated meiogynin A from the bark of *Meiogyne cylindrocarpa*, a Malaysian plant, and demonstrated it to be a Bcl‐x_L_ inhibitor.^58^ Subsequently, in 2014 Roussi and co‐workers reported the preparation of a number of meiogynin A derivatives, designed by a structure activity relationship study, which was reported at the time to be “one of the most potent dual inhibitors” of Mcl‐1 and Bcl‐x_L_.^59^ The most effective compound, titled Roussi compound 2a, possessed *K*_i_ values of 106 nm for Mcl‐1 (Bid‐BH3 only FP assay) and 153 nm for Bcl‐x_L_ (Bak‐BH3 only FP assay). In addition, the selective Mcl‐1 inhibitor Roussi compound 2c showed *K*_i_ values of 460 nm for Mcl‐1 (Bid‐BH3 only FP assay) and >23 μm for Bcl‐x_L_ (Bak‐BH3 only FP assay).

### 3.12 Sabutoclax


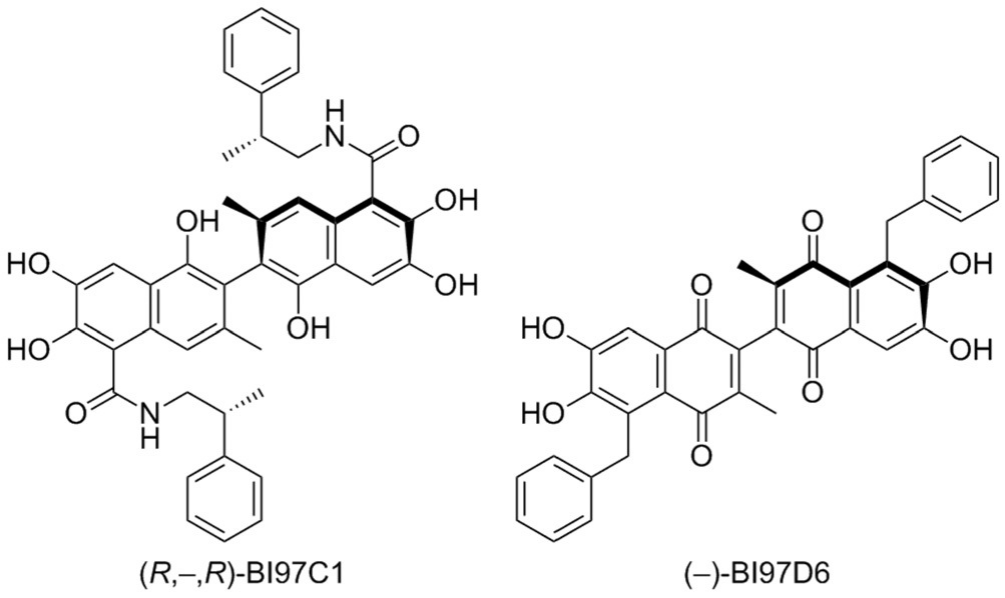
In 2009 Pellecchia and co‐workers examined the derivatisation of gossypol guided by NMR binding assays and computational docking.^60^ From the library of compounds Pellecchia prepared, racemic BI97C1 (originally compound 8r) was the most effective, demonstrating IC_50_ values of 0.28 μm for Mcl‐1 and 0.32 μm for Bcl‐2 (FITC‐Bak‐BH3‐only peptide), as well as binding to Bcl‐x_L_ and Bfl‐1. Additionally, Bl97C1 was shown to induce apoptosis in the RS11846 human lymphoma cell line. In a follow up publication, Pellecchia et al. examined the stereochemistry of Bl97C1, demonstrating that the (R,‐,R) enantiomer (displayed) inhibits the binding of BH3 peptides to Bcl‐x_L_, Bcl‐2, Mcl‐1, and Bfl‐1 with IC_50_ values of 0.31, 0.32, 0.20, and 0.62 μm (FITC‐Bak‐BH3‐only peptide).^61^ Additionally, inhibition of the cell growth of human prostate cancer, lung cancer, and BP3 B‐cell lymphoma cell lines with EC_50_ values of 0.13, 0.56, and 0.049 μm, respectively was demonstrated.

Following this Pellecchia and co‐workers examined the elaboration of apogossypolone in a similar manner as gossypol.^62^ The study highlighted racemic BI97D6 as a potential candidate, demonstrating inhibition of Bcl‐x_L_, Bcl‐2, Bfl‐1, Mcl‐1 at IC_50_ values of 0.34 μm, 0.29 μm, 0.65 μm, and 0.24 μm, respectively (FITC‐Bak‐BH3‐only peptide). Pellecchia again followed this with an examination of the stereochemistry, highlighting (−)‐BI97D6 (displayed) as a potent binder. (−)‐BI97D6 demonstrated binding inhibition of Bcl‐x_L_, Bcl‐2, Mcl‐1, and Bfl‐1 with IC_50_ values of 76, 31, 25, and 122 nm, respectively in FP assays (FITC‐Bak‐BH3‐only peptide).^63^ Additionally, (−)‐BI97D6 inhibited growth of the PC‐3 human prostate cancer and H23 human lung cancer cell lines with EC_50_ values of 0.22 and 0.14 μm, respectively. Subsequently, Andreeff and co‐workers demonstrated that (−)‐BI97D6 overcomes ABT‐737 resistance in acute myeloid leukaemia.^64^

### 3.13 Marinopyrrole A derivatives


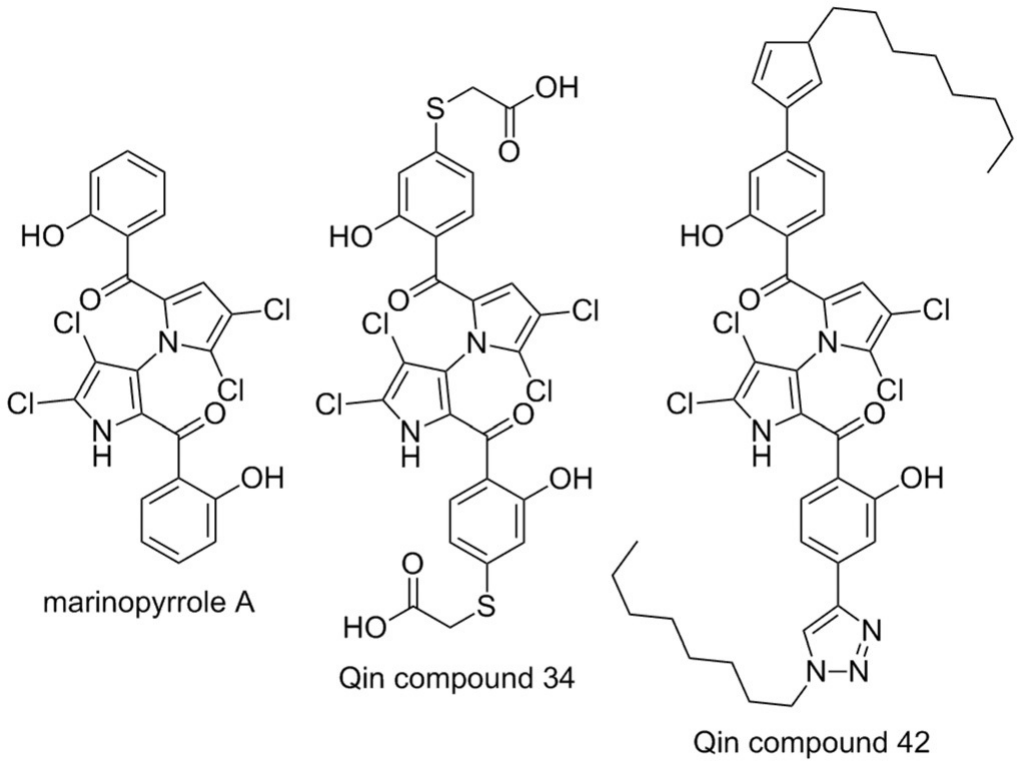
Marinopyrrole A is a natural product antibiotic isolated from a *Streptomyces* sp. reported in 2009.^65^ It was described by Wang and co‐workers to possess the ability to overcome ABT‐737 resistance by inhibiting the action of Mcl‐1.^66^ However, it was subsequently reported to have no effect on Mcl‐1 in cells, and exhibits the same action on Bcl‐2‐dependent cells (2 μm) as Mcl‐1‐dependent cells (2.5 μm).^67^ In 2015 Qin and co‐workers reported the preparation of a number of analogues of marinopyrrole A, following a structure activity relationship study.^68^ The talismanic compounds of this study were titled Qin compound 34, which showed 16 fold selectivity for Mcl‐1 (IC_50_=6.1 μm, Bim‐BH3‐only peptide, ELISA) compared with Bcl‐x_L_ (IC_50_> 100 μm, Bim‐BH3‐only peptide, ELISA), and Qin compound 42, which showed potent activity for both Mcl‐1 and Bcl‐x_L_ (IC_50_=0.6 μm, 0.5 μm respectively, Bim‐BH3‐only peptide, ELISA). However despite being highly potent in the ELISA, compound 42 had little activity in intact breast cancer cells.

### 3.14 Chai compounds 6 & 7


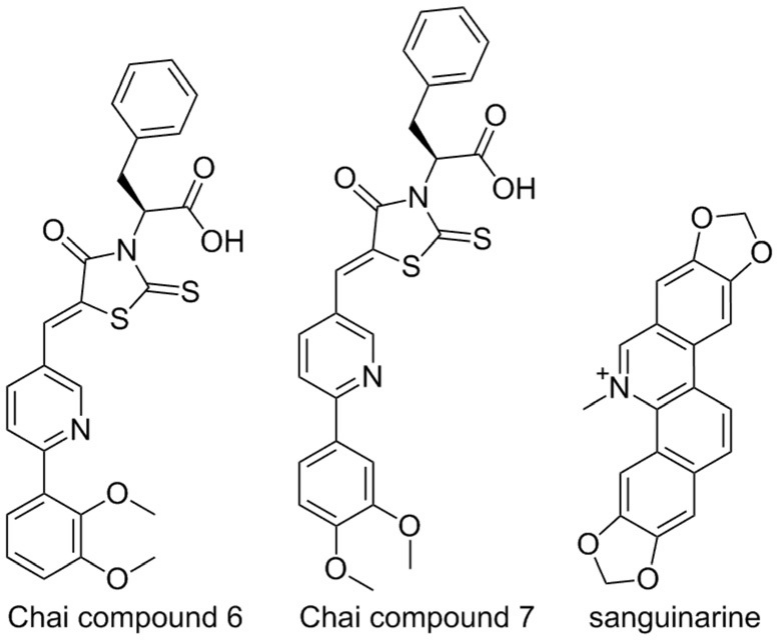
In 2010 Chai and co‐workers addressed the problem of identifying a selective Mcl‐1 inhibitor.^69^ All Mcl‐1 inhibitors previously reported had been pan‐Bcl‐2 inhibitors, with moderate to weak activity, making overcoming Mcl‐1 induced resistance a challenge. Chai screened a library of compounds which incorporated known Bcl‐2 family binders BH3I‐1 and sanguinarine with a FP assay using Mcl‐1 and Bak.^70^ This screening highlighted two compounds, referred to in the original literature as compounds 6 and 7. These constitutional isomers showed significant differences in selectivity, with Chai compound 6 displaying binding in the micromolar range to both Bcl‐x_L_ (*K*_i_=3.7 μm) and Mcl‐1 (*K*_i_=6.9 μm), and Chai compound 7 showing selectivity toward Mcl‐1 (*K*_i_=8 μm) with no binding to Bcl‐x_L_ (*K*_i_> 100 μm, Flu‐Bak‐BH3 peptide competitive binding).^69^ Both compounds showed greater binding in their assays than BH3I‐1, with NMR studies demonstrating that the compounds were binding in the BH3 domain. Docking studies demonstrated that the Mcl‐1 binding groove is wider than that of Bcl‐x_L_, which may explain the selectivity being displayed by the two constitutional isomers.^69^

### 3.15 MIM1


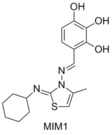
In 2010 Walensky and co‐workers showed that the BH3 domain of Mcl‐1 was a potent and selective natural inhibitor of Mcl‐1.^25^ This prompted the use of the fluorescently tagged BH3 domain of Mcl‐1 as the competitive binding agent for their FP assays, allowing for a selective and potent Mcl‐1 binder to be identified. 71 296 compounds were screened for the ability to displace a FITC tagged Mcl‐1 BH3 domain peptide, and stringent selection processes highlighted MIM1 as a potent and selective Mcl‐1 binder.^27^ MIM1 displaced the FITC‐Mcl‐1‐BH3 peptide at an IC_50_ of 4.7 μm, but had no significant ability to displace Bid from Bcl‐2, complementing the activity of ABT‐737. The ability of MIM1 to induce apoptosis in Mcl‐1‐dependent leukaemia cells was also demonstrated.

### 3.16 Cardone compound 9


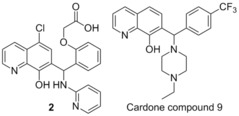
Researchers at Eutropics Pharmaceuticals performed a high throughput screen of 315 000 compounds, searching for targets which inhibit Mcl‐1, but do not bind to Bcl‐x_L_. This screen highlighted a 7‐hydroxyquinoline **2**, displaying inhibition of Mcl‐1 with an IC_50_=2.4 μm, with no inhibition of Bcl‐x_L_(competitive FP assay with FITC‐Bim BH3‐only peptide).^71^ Structure activity relationship studies of this compound resulted in the preparation of compound 9 which displayed improved affinity for Mcl‐1 (IC_50_=0.31 μm) with relatively low inhibition of Bcl‐x_L_ (IC_50_>40 μm) (FITC‐Bim BH3‐only peptide). Cardone compound 9 also showed moderately high liver microsome stability, with a half‐life of 55 min in human microsomes, and computational studies demonstrated that it was a BH3 domain binder. Cardone and co‐workers showed that Cardone compound 9 had high activity in a panel of human derived cancer cell lines, in particular Mcl1‐1780 (EC_50_=0.3 μm), Bcl2‐1863 (EC_50_=1.1 μm), NCI‐H929 (EC_50_=1.6 μm) and SUDHL‐6 (EC_50_=3.3 μm). This cytotoxicity was shown to be a result of apoptosis via Annexin V staining, demonstrating a complementary activity to ABT‐263 in regards to cell lines expressing high amounts of Bcl‐2/Bcl‐x_L_ and Mcl‐1.^71^

### 3.17 Tanaka compound 11


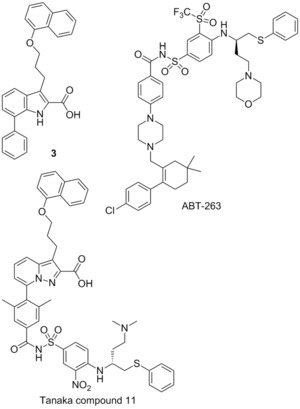
In an attempt to overcome the ability of Mcl‐1 to induce resistance to the compounds which target Bcl‐2 and Bcl‐x_L_ undergoing clinical trials, Tanaka and co‐workers envisaged a chimeric compound which used elements from known Mcl‐1 binders and Bcl‐x_L_ binders.^72^ In this manner they used ABT‐263 (which was in clinical trials at the time)^73^ and the known Mcl‐1 binder 3, to develop a potent inhibitor of both Mcl‐1 and Bcl‐x_L_, originally termed compound 11. Tanaka′s compound 11 demonstrated IC_50_ values of 88 nm for Mcl‐1 and 3.7 nm for Bcl‐x_L_ (Bid‐BH3 only, time‐resolved fluorescence resonance energy transfer assay).

### 3.18 Fesik fragment‐based screening


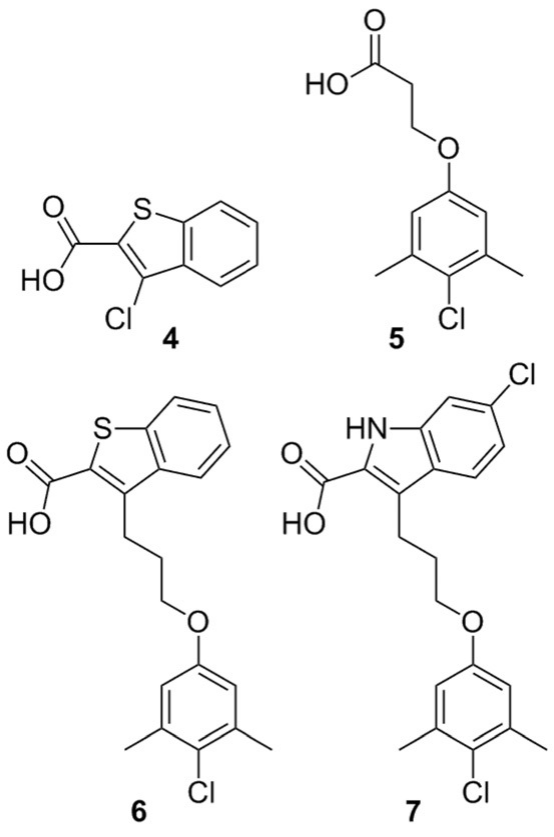
In 2013 Fesik and co‐workers described the use of a now commonly employed technique to target protein–protein interactions. They screened small fragments for micromolar binding, and then using NMR studies identified the binding sites of each fragment. With this information in hand each fragment can be linked together to create a small molecule which possesses greater binding affinity. In this way the relatively large protein–protein binding site can be explored rapidly to identify the important binding motifs.^74^ Employing this method, Fesik was able to identify two important regions, which were bound to by distinct small molecules, exemplified by **4** (*K*_i_=131 μm) and **5** (*K*_i_=60 μm, FITC‐Mcl‐1‐BH3‐only peptide). Merging these two fragments generated **6** (*K*_i_=0.32 μm, FITC‐Mcl‐1‐BH3‐only peptide), which was confirmed by crystallography to occupy both of the pockets identified by the initial fragments. Subsequently this molecule underwent structure‐guided synthetic design, which yielded several high‐affinity leads, exemplified by **7** which showed a *K*_i_=0.055 μm, with 10‐fold selectivity for Mcl‐1 over Bcl‐2 and Bcl‐x_L_ (FITC‐Mcl‐1‐BH3‐only peptide). Fesik also described several other potential leads with the same skeleton.^74^

### 3.19 Benzylpiperazine derivatives


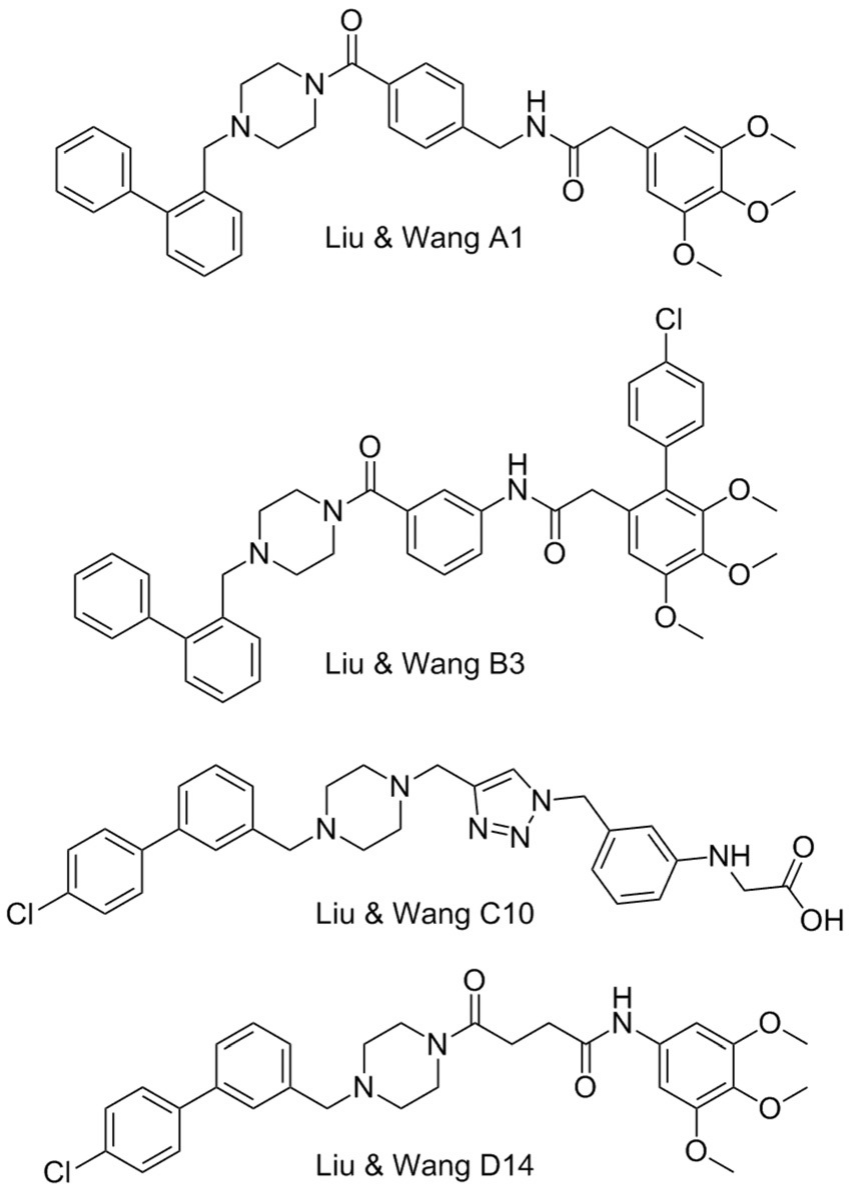
In 2013 Liu, Wang, and colleagues reported the use of an innovative computational modelling approach to develop four molecular scaffolds for synthetic evaluation.^75^ Liu and Wang apply the assumption that critical binding residues of the PPI exist in small clusters, which can be exploited by small fragments. Using computational docking, one can examine these small clusters to identify the most appropriate fragments, and then combine these virtually, with the appropriate chemical spacing to create molecular scaffolds. In this manner Liu and Wang developed four backbones, termed series A‐D, exemplified by the best performers in each series, A1, B3, C10 and D14. The A and D series were found to outperform series B and C in their FP binding assay, with A1 demonstrating a *K*_i_=0.18 μm and D14 showing a *K*_i_=0.32 μm (Mcl‐1/5‐FAM‐Bid BH3‐only peptide competitive binding assay). These compounds also showed some selectivity for Mcl‐1, with A1 showing no appreciable binding to Bcl‐2 or Bcl‐x_L_ (5‐FAM‐Bid BH3‐only peptide).^75^

### 3.20 UMI‐77


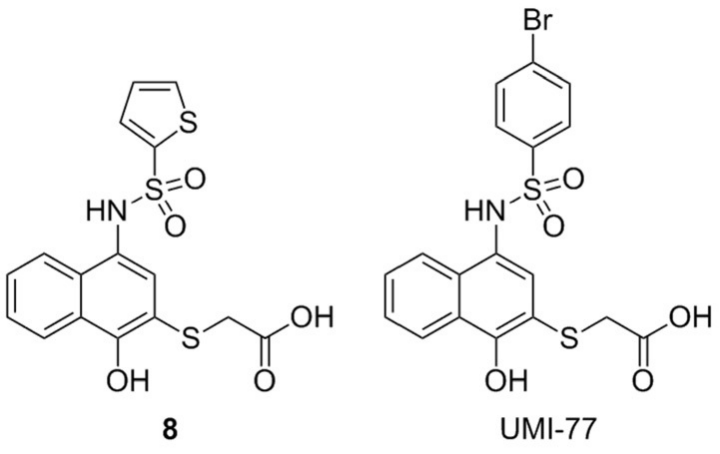
In 2014 Nicolovska‐Coleska performed a high‐throughput screen to identify small‐molecule inhibitors of Mcl‐1.^76^ They identified compound **8** which led to the development of UMI‐77 through structure‐based chemical modifications. With FP‐based screens Nicolovska‐Coleska showed that UMI‐77 displaces Bid‐BH3 from Mcl‐1 with a *K*_i_=0.49 μm, with a 10‐fold selectivity, with Bcl‐w, Bcl‐2, and Bcl‐x_L_ displaying *K*_i_>5 μm (5‐FAM‐Bid BH3‐only peptide). It was also demonstrated that UMI‐77 selectively binds to Mcl‐1 in preference to Noxa in a dose‐dependent manner, by binding to the BH3‐binding pocket of Mcl‐1. In cell lines, UMI‐77 was shown to inhibit the growth of pancreatic cancer cells (MiaPaCa‐2 and AsPC‐1) and showed single‐agent antitumour activity toward BxPC‐3 cells. Morgan and co‐workers then demonstrated that UMI‐77 radio‐sensitised cancer cell lines BxPC‐3 and Panc‐1, but did not radio‐sensitise normal small intestinal cells.^77^ Morgan demonstrated that ABT‐737, which does not bind to Mcl‐1, did not perform in the same manner, suggesting Mcl‐1 plays a key role in radio‐sensitising cancer cells.

### 3.21 A‐1210477


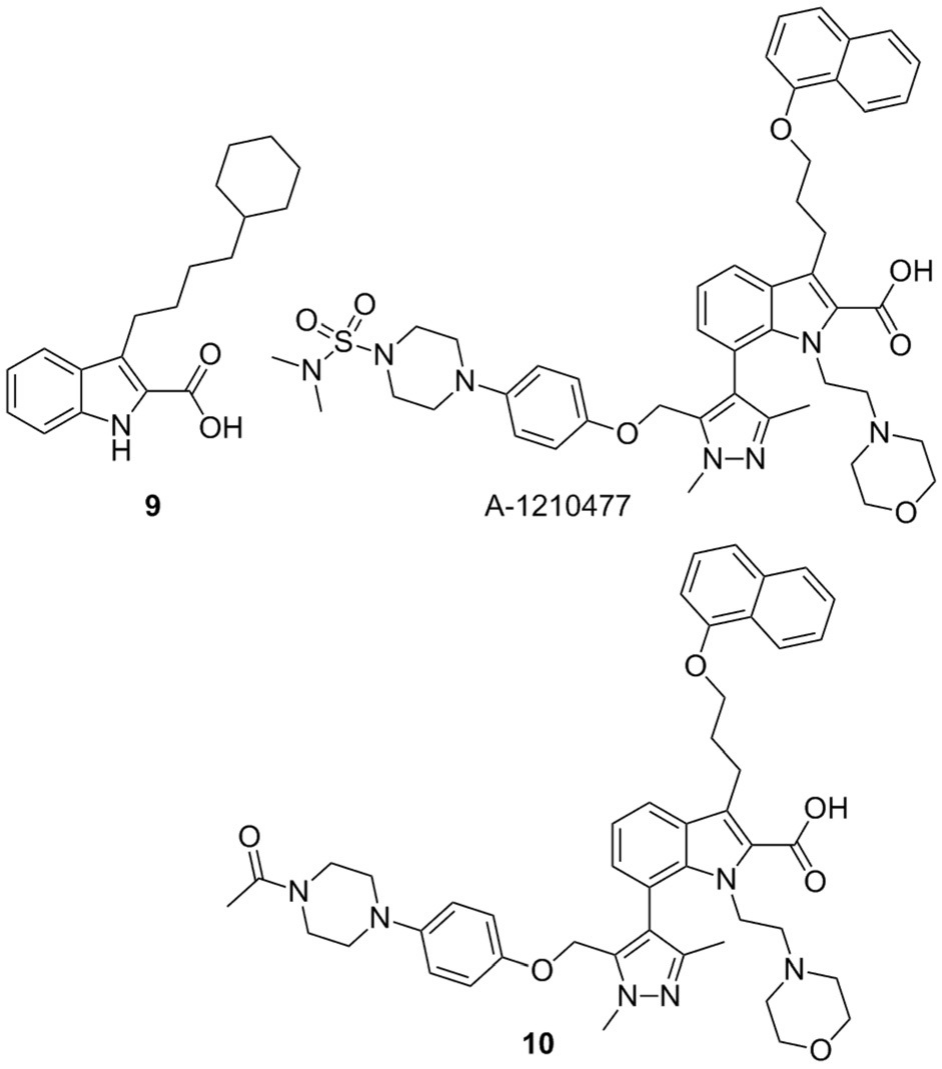
Souers and co‐workers recently identified **9** through a high‐throughput screen aimed at identifying small‐molecule inhibitors of Mcl‐1 using the selective protein Noxa.^78^ **9** showed micromolar affinity for Mcl‐1 (*K*_i_=4.4 μm), as well as selectivity over Bcl‐x_L_ (*K*_i_>10 μm), in an FP assay. Structure guided design, incorporating hits which they had generated in previous reports,^79^ allowed for the preparation of A‐1210477, which showed a 10 000‐fold improvement in affinity for Mcl‐1 (*K*_i_=0.43 nm, Noxa BH3‐only peptide) compared to **9**. A‐1210477 also showed similar selectivity for Mcl‐1 with *K*_i_>0.66 μm for Bcl‐2, Bcl‐x_L_, Bcl‐w and A1. Activity was proven to be through the disruption of the Mcl‐1 Bim complex in live cells. A‐1210477 was found to induce apoptosis in the cancer cell line H929 and restore navitoclax sensitivity in the resistant pancreatic cell line BxPC‐3.^80^ Similarly in the breast cancer cell line SKBR3 sensitivity to navitoclax was restored with the addition of A‐1210477 resulting in the release of BAK, demonstrating the role of Mcl‐1 in navitoclax resistance.^81^ The molecule is believed to bind to Mcl‐1 in a similar manner to analogue **10** reported in the same manuscript (where the sulfonamide on the piperazine was replaced with an acetyl group); specifically binding in the BH3 binding domain and mimicking the BIM BH3 peptide. The central indole binds in the p2 pocket, with the carboxylate group forming a hydrogen bonding interaction with Arg263. The naphthyl group was projected toward the p1 pocket and the extended piperazine spanned the p3/p4 pockets. A‐1210477 represents the most potent small‐molecule inhibitor described in the literature to date, and induces clear on‐target cellular activity.

### 3.22 Pyridoclax


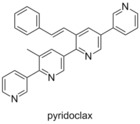
In 2015 Voisin‐Chiret and Poulain employed a strategy similar to that which lead to the discovery of BH3M6.^82^ Examining the binding pocket of Mcl‐1, and its differences from the other Bcl‐2 family members, led them to employ an oligopyridine backbone to act as a BH3‐mimetic. Computational modelling and SAR studies resulted in the preparation of pyridoclax, which was designed based on the binding of the selective Mcl‐1 binding protein Noxa. Using bioluminescence resonance energy transfer (BRET) it was shown that pyridoclax binds to Mcl‐1 in the cell (Bim/Mcl‐1), with a maximum effect observed at 25 μm. Subsequently it was demonstrated that pyridoclax sensitises various cancer cells (IGROV1, OAW42‐R, SLOV3, A549 and MSTO‐211 H) to Bcl‐x_L_ knock‐down, and chemoresistant ovarian cancer cells to ABT‐737 (IGROV1‐R10 and SKOV3) at 25 μm, suggesting Mcl‐1 selectivity.

## 4 Summary and Outlook

The discovery of chemical entities capable of modulating PPIs is a challenging prospect and PPIs have historically been described as “undruggable”. However, the efforts of many research groups over the past two decades has defied this view point with a number of inhibitors progressing through clinical trials which target the Bcl‐2 family (ABT‐737, navitoclax and ABT‐199).^73^, ^83^ However, despite the recent success in targeting Bcl‐2 and Bcl‐x_L,_ the modulation of Mcl‐1 with inhibitors has proven elusive and there are currently no Mcl‐1 inhibitors in clinical trial despite the fact that Mcl‐1 is one of the most commonly amplified genes in cancer.^84^

Perhaps more promisingly, recent years have seen an increase in interest in targeting Mcl‐1 and identifying new inhibitors—both small‐molecule and peptide based. Specifically, stapled peptides are a promising new approach to developing moieties capable of inhibiting PPIs and the examples described above by Walensky^20^ and Lin^28^ demonstrate the potential of this class of therapeutic. Through fixing the conformation of the α‐helix, the active conformation of the peptide is maintained leading to an increase in affinity for the protein. In addition, stapling the peptide can shield the amide bonds of the peptides protecting them from proteases and also decrease their ionic character, resulting in an improvement in cellular uptake (through passive diffusion) and reduced clearance from the body. With the number of publications reporting stapled peptides accelerating (just one in 2000 vs 16 in 2013) and the first clinical trial successfully completed in 2013, the clinical potential of stapled peptides is currently being explored but it remains to be seen if this will translate into the clinic and if stapled peptides are able to fulfil this potential.

The number of publications describing small‐molecule inhibitors of Mcl‐1 has increased rapidly in recent years. However, a large proportion of the small molecules described have been identified through high throughput screening of larger libraries, resulting in large attrition rates. In addition achieving selectivity has proved problematic due to the very subtle differences in binding pockets between the Bcl‐2 family; the p2 pocket of Mcl‐1 is dynamic compared to Bcl‐2, whereas the p4 pocket is less well defined, shallower and less hydrophobic than Bcl‐x_L._ 85 In fact most of the early compounds reported were pan Bcl‐2 inhibitors with the first selective Mcl‐1 inhibitor reported in 2010.^69^ There is also a lack of in vivo data for the majority of compounds reported in the literature, although this has been addressed very recently by A‐1210477, where the authors argue this is the first description of a small molecule with sufficient potency to have a clear on‐target cellular effect.^80^

Despite these challenges, advances have been made and the outlook is promising for the discovery of Mcl‐1 inhibitors. New methodologies are being employed to target PPIs which are proving effective and increasing the hit rate of target‐based screening. The computational approach employed by Liu and Wang decreases screening time significantly.^75^ The fragment‐based approach employed by Fesik allows for smaller fragment libraries to be evaluated which quickly generates information about effective ways to target the binding grooves of the PPI.^74^ The recent work of Souers provides a positive outlook, with potent and selective Mcl‐1 inhibitors being developed through the use of selective assays.^78^, ^79^ The exploitation of a combination of these techniques may allow for the highly efficient development of drug candidates. The examples of Mcl‐1 inhibitors described here demonstrate that the “undruggable” challenge that PPIs present can be tackled effectively and is likely to lead to a novel inhibitors for the treatment of cancer.
